# Lattice Energy Partitions in Crystals of Flexible Molecules and the 40% Limit

**DOI:** 10.1021/jacs.5c09997

**Published:** 2025-10-15

**Authors:** Amrita Chattopadhyay, Adam R. Hill, Sarah E. Wright, Gregory J. O. Beran, Aurora J. Cruz-Cabeza

**Affiliations:** † Department of Chemistry, 3057 Durham University, Durham DH1 3LE, U.K.; ‡ Department of Chemistry, 5292 The University of Manchester, Manchester M139PL, U.K.; § Department of Chemical Engineering, The University of Manchester, Manchester M139PL, U.K.; ∥ Department of Chemistry, 8790 University of California Riverside, Riverside, California 92521, United States

## Abstract

Flexible molecules change their conformations to optimize intermolecular interactions in the solid-state —a process that comes at an intramolecular energy cost. The interplay between these opposing forces leads to beautifully balanced and elegant crystal structures. Yet, the relationship between intramolecular energy penalties and intermolecular stabilization has not been fully explored, largely because it requires models capable of accurately capturing components with distinct physical origins. In this work, we employ state-of-the-art hybrid DFT models to probe these interactions and quantify their energetic contributions to crystal lattice energies. After benchmarking 18 computational methods, we identify PBE-MBD/B2PLYPD as the method best reproducing experimental polymorph stabilities with mean absolute deviations of just 2.3 kJ·mol^–1^ across 17 polymorphic pairs. Using this model, we compute and analyze lattice energy partitions for 125 crystal structures spanning a diverse set of flexible compounds. Our analysis reveals a striking empirical trend—the “40% limit”—which shows that up to 40% of the intermolecular stabilization energy can compensate for intramolecular energy penalties associated with conformational variations. Furthermore, the probability of a higher-energy conformation being observed in the solid-state decreases as a function of the intra-to-intermolecular energy ratio and becomes negligible at the 40% mark. These findings are significant: they define the energetic limits of conformational flexibility in the solid-state and the intra-to-intermolecular energy ratios provide a quantitative tool to predict structures. They can be used to guide the conformational phase space sampling for crystal structure prediction, to rank hypothetical structures according to their crystallizability, or to anticipate difficulties of nucleation and crystal growth in crystal forms of flexible compounds.

## Graphical abstract

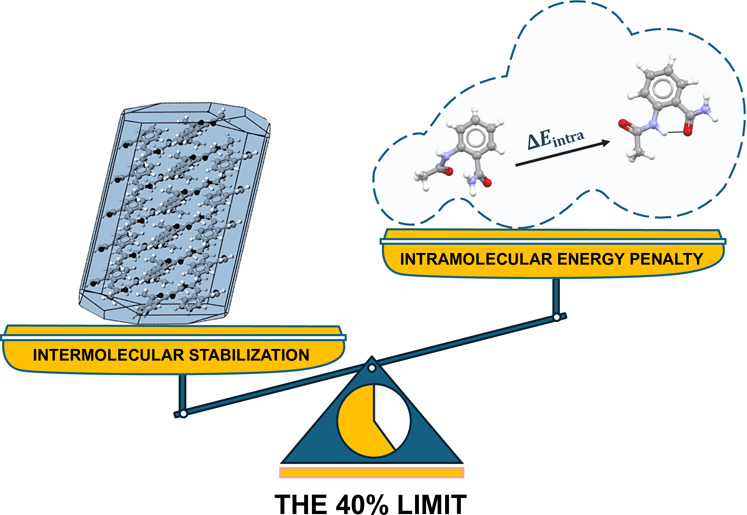

## 1 Introduction

Over 100 million chemical compounds have now been synthesized, with the plausible chemical space extending into the billions.^1^ A common feature among most compounds is molecular flexibility—the ability of molecules to adopt multiple three-dimensional shapes (conformations) via bond rotations. This flexibility is fundamental to molecular function and properties. For instance, bioactivity often depends on specific conformations that interact with protein receptors,^2^ while dynamic conformational changes can enable molecular motion.^3^ In the solid-state, molecular flexibility influences phenomena such as conformational polymorphism,^4^ the formation of solvates and cocrystals,^5^ or the dynamic behavior of crystalline materials.^6^

In pharmaceuticals, nearly all drug molecules (and especially modern drugs) exhibit significant conformational flexibility (Figure 1a), and their solid-state conformations directly impact drug delivery and performance. Understanding and controlling how flexible molecules crystallize is therefore critical to drug development and manufacturing. While crystallization screening and computational modeling are routinely employed in pharmaceutical pipelines, a key challenge remains: how do molecules balance intramolecular and intermolecular interactions in the crystal-state and during crystallization?

**Figure 1:**
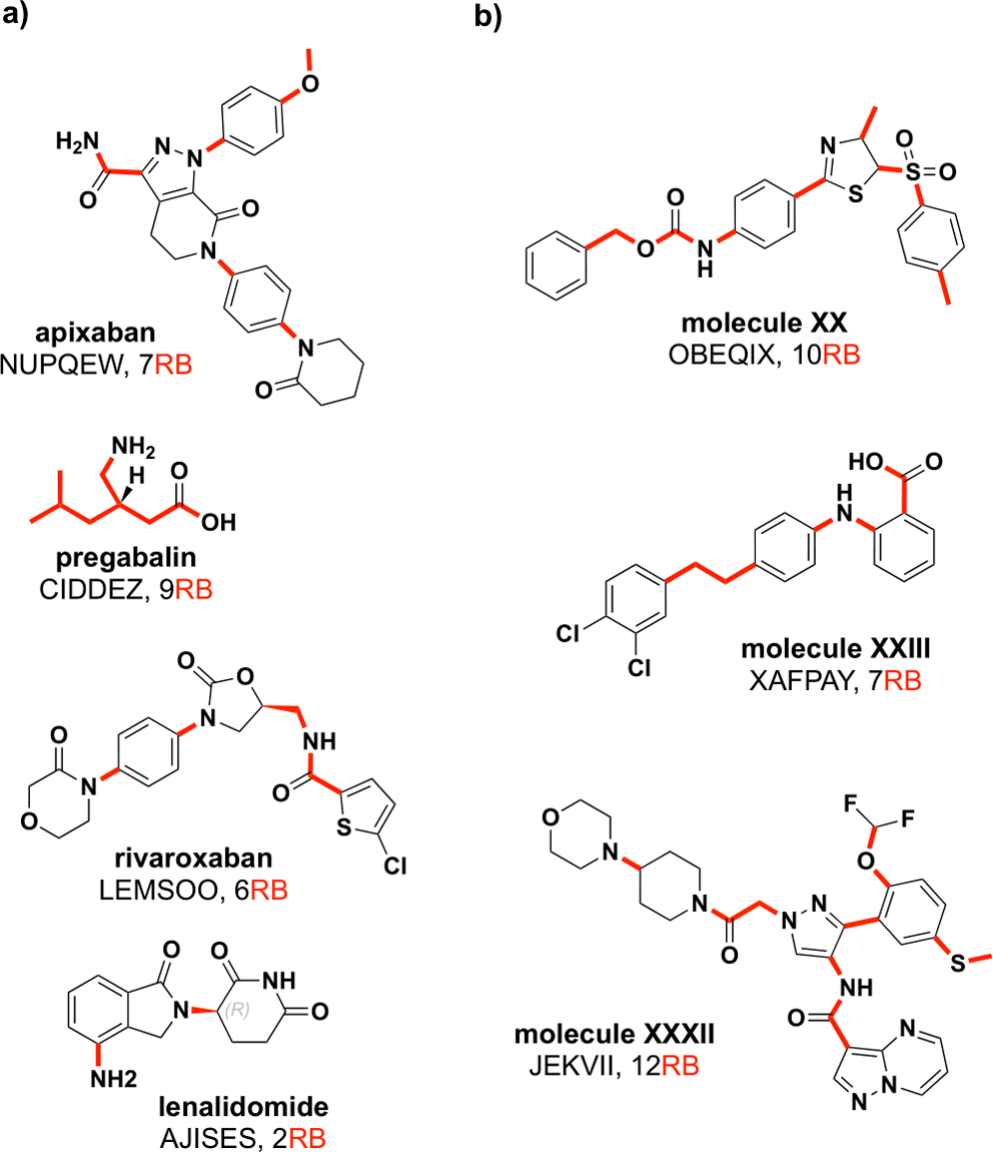
Structures of four of the top-selling small molecule drugs (a) and Molecules XX, XXIII, and XXXII from the fifth, sixth, and seventh Blind Test for crystal structure prediction (b). Rotatable bonds are highlighted in red.

The packing of flexible molecules in the solid-state is inherently complex, as each conformation can be packed into thousands of distinct hypothetical crystal structures. Over the past 25 years, computational Crystal Structure Prediction (CSP) of molecular crystals has become a reality^7–10^ even for compounds of molecular complexity typical of pharmaceuticals (Figure 1b).^11^ However, CSP of flexible molecules remains computationally demanding due to two primary factors: (a) the high dimensionality of the search space, with each rotatable bond introducing an independent variable,^12,13^ and (b) the need for accurate energy ranking models that account for both electron delocalization^14–16^ and dispersion interactions.^17–19^ As a result, predicting the crystal structures of large and flexible molecules requires substantial computational resources. For example, independent CSP landscapes for molecule XXXII in the seventh Blind Test consumed between 600,000 and nearly 4 million CPU hours each.^20,21^ To mitigate these costs, machine learning force fields are increasingly employed to accelerate the search stage of CSP.^22,23^ Alongside these technological developments, a deeper scientific understanding of how flexible molecules pack in the solid-state,^24^ their energetics^25^ and polymorphism^4,26^ is essential.

Crystallization itself—typically from solution in pharmaceutical contexts—is even less well understood for flexible molecules. In solution, molecules can adopt a wide range of conformations, which may differ from those found in the solid-state. This conformational diversity can hinder nucleation and growth, as well as lead to the formation of multiple crystal forms.^27^ Moreover, molecular flexibility has been linked to crystallizability, with large flexible molecules often failing to crystallize^28^ or requiring extensive experimental efforts to enable them to do so.^29^ For flexible compounds, the adoption of the “correct” conformer for crystallization is a critical step,^30–34^ and recent work has shown that “incorrect” solution conformations can even lead to self-poisoning during crystal growth.^35^

In this work, we investigate the relationship between intramolecular and intermolecular energies in crystals of flexible molecules to better understand this balance and its consequences for modeling and crystallization. First, we benchmark 18 state-of-the-art computational models to identify the best-performing model to accurately reproduce experimental lattice energies. Using the best-performing model, we then calculate and analyze lattice energy partitions in 125 crystals of flexible molecules and uncover key trends that have important implications for CSP and crystallization.

## 2 Results and Discussion

### 2.1 Systems for Benchmarking

We selected a benchmarking data set comprising 15 flexible compounds, each exhibiting polymorphism, for which experimental data on the relative stabilities of 17 polymorphic pairs were gathered from the literature (Figure 2). This data set was divided into two subsets: (a) the validation subset with 6 compounds forming 7 polymorphic pairs and (b) the test subset with 9 compounds forming 10 polymorphic pairs. The validation subset was used to assess the performance of 18 computational methods (see Section 2.2), enabling the identification of the most accurate approach, referred to as the Best Lattice Energy Model (BLEM). Once selected, the BLEM was applied to the test subset to evaluate its predictive accuracy.

**Figure 2:**
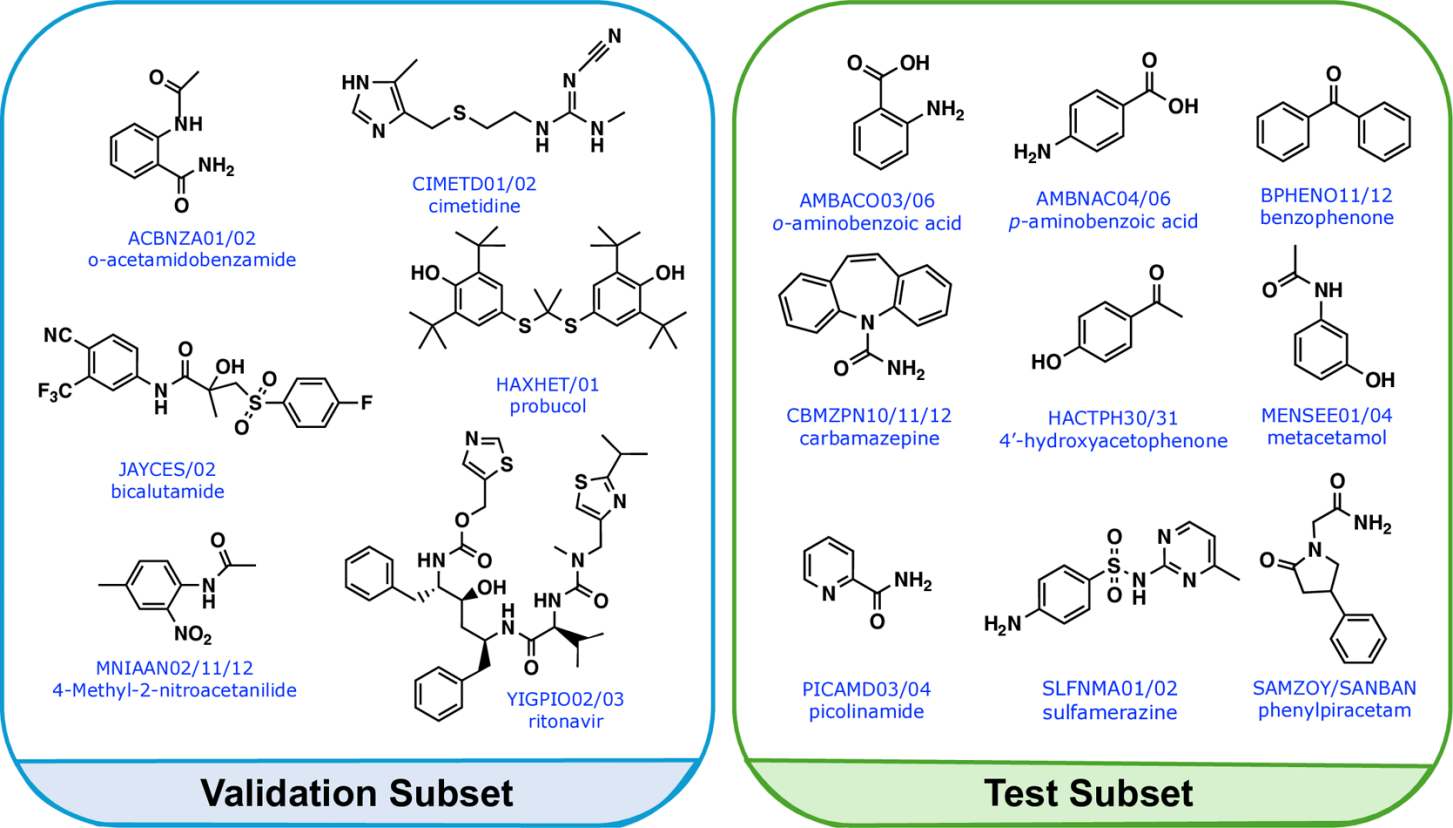
Benchmarking set of polymorphic systems used in this study, divided into validation and test subsets.

The compounds included in our benchmarking data set exhibit considerable diversity in both size and structural complexity, with all systems being conformationally flexible. The validation subset consists exclusively of conformational polymorphs sourced from the Cambridge Structural Database (CSD),^36^ for which distinct crystal conformations and significant differences in intramolecular energies have been previously documented.^4,25^ This makes the subset particularly well-suited for validation, as it challenges computational models to accurately capture the delicate balance between intra- and inter-molecular interactions—an essential requirement for the purpose of the present work.^25^ Moreover, several compounds in the validation subset are relevant drug compounds, including probucol and ritonavir. The test subset comprises additional polymorphic pairs with reported experimental stabilities, providing a robust basis for evaluating the predictive performance of the selected BLEM.

Experimentally, relative stabilities of polymorphs are typically determined via thermal analysis, either by comparing their heats of fusion or through direct observation of polymorphic transitions. The resulting differences in experimental enthalpies (Δ*H*_exp_), usually measured at temperatures between 393 and 433 K, can be directly compared to computed lattice energy differences at 0 K. This comparison is justified by the typically negligible differences in heat capacities between polymorphs.^37^

### 2.2 Energy Terms and Energy Models

A key objective of this study was the computation and analysis of the partitioned components of lattice energies in crystals of flexible compounds. To achieve this, the total lattice energy was decomposed into two distinct contributions (eq 1): an intermolecular energy term (*E*_inter_), which arises from molecule–molecule interactions within the crystal lattice, and an intramolecular energy term (*E*_intra-global_), which represents the energetic penalty associated with conformational distortion required to optimize crystal packing.

This intramolecular energy can be further resolved into two subcomponents: the adjustment energy (*E*_adjustment_), also referred to as strain energy, and the global change energy.^4^ The adjustment energy accounts for the energy required to distort a gas-phase conformer into the conformation observed in the crystal. In contrast, the global change energy captures the difference in energy between the gas-phase conformer leading to the crystal conformation relative to the most stable gas-phase conformer (Δ*E*_change‑global_). Identifying the global minimum gas-phase conformer requires a conformational search, which in our study was performed using the CSD Conformer Generator, as detailed in the Methods section. Several other computational tools are available for exploring conformational landscapes and have been previously employed for similar analyses.^38,39^

1 $$E_{latt‐global}=E_{inter}+E_{intra‐global}=E_{inter}+\left(E_{adjustment}+\Delta E_{change‐global}\right)$$

Intermolecular contributions to the lattice energies were calculated using two dispersion-corrected density functional theory (DFT) methods: PBE-TS and PBE-MBD.^25^ While we also attempted to use PBE0-MBD for the computation of intermolecular energies (since it has been proven to perform very well),^17,40,41^ this method was found to be too computationally demanding for some of our systems (e.g., ritonavir) and hence was only used for intramolecular energy calculations. Intramolecular energy components were evaluated using a set of nine DFT functionals, including the generalized gradient approximation (GGA), hybrid, or double-hybrid functionals PBE-TS, PBE-MBD, PBE0-MBD, *ω*B97M-V, M06-D3, M062X-D3, revDSD-PBEP86-D4, *ω*B97X-D and B2PLYPD. By systematically combining each intermolecular with each intramolecular method, a total of 18 distinct computational approaches were generated and validated against the experimental data. All calculations were performed at 0 K since the purpose of the work was to study intramolecular to intermolecular energy contributions. The computation of finite temperature effects was beyond the scope of this work.

### 2.3 Selection and Test of the Best Lattice Energy Model (BLEM)

Computed lattice energy differences between polymorphs in the benchmark validation subset were compared with experimental values and mean absolute deviations (MADs) calculated for the 18 computational models considered (Figure 3). The energy models are arranged from lower to higher accuracy, with intermolecular model types shown in gray (PBE-TS) and red (PBE-MBD), and the intramolecular models indicated along the *x*-axis. Across all intermolecular models, the PBE-MBD intermolecular treatment consistently yielded lower MADs than PBE-TS, highlighting the importance of many-body dispersion corrections for accurate lattice energy calculations—consistent with previous findings.^17,19^ Among the intramolecular models, the B2PLYPD double hybrid functional provided the best agreement with experiment, achieving a MAD of just 2.3 kJ/mol. This aligns with prior studies emphasizing the need to account for electron correlation when modeling crystals of flexible molecules.^14,15,42–44^ The second-best intramolecular energy model, *ω*B97XD, also performed well with a MAD of 2.4 kJ/mol.

**Figure 3:**
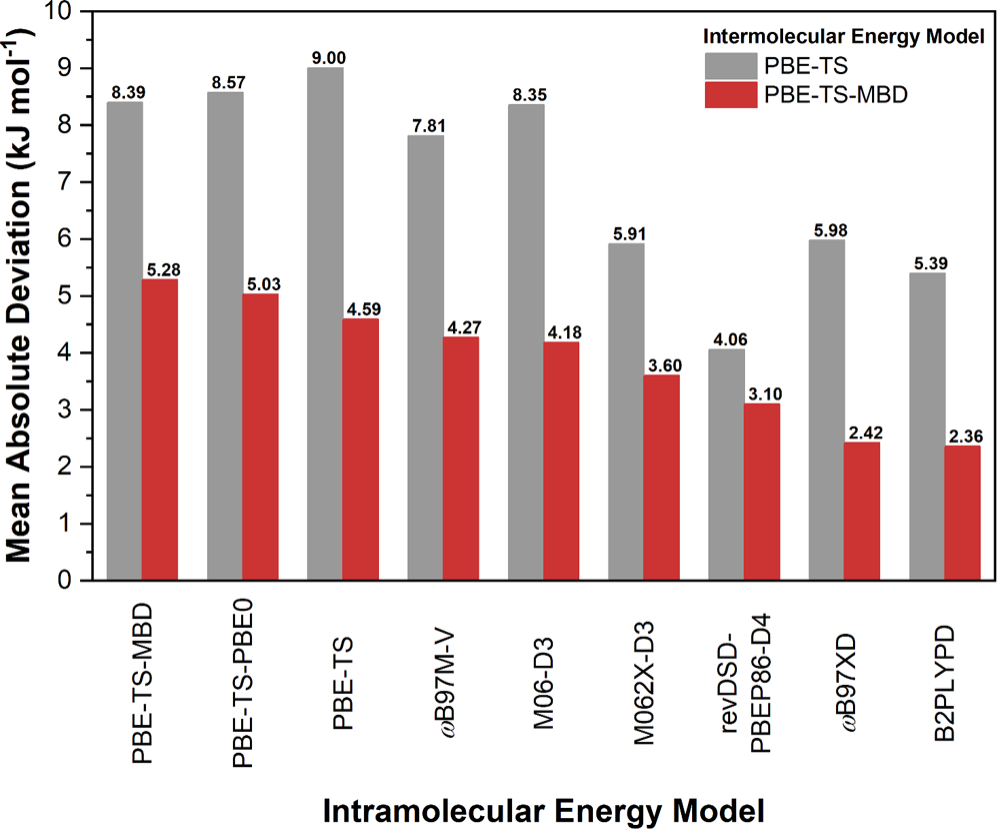
Mean absolute deviation between predicted relative lattice energies and experimental lattice enthalpies for the benchmarking validation subset as a function of the energy model.

Consequently, we selected the inter/intra-molecular BLEM model PBE-MBD/B2PLYPD to compute the relative lattice energies of the polymorphic systems in the benchmarking test subset. In agreement with the validation subset, the BLEM yielded a MAD of just 2.3 kJ/mol, reaffirming the accuracy and reliability of our method.

Table 1 presents the computed versus experimental energy differences for all systems in the full benchmarking set, divided into the validation and test subsets. The predicted stabilities from our BLEM model show excellent agreement with experimental thermal lattice enthalpies, achieving an overall MAD for the entire benchmarking set of 2.3 kJ/mol across all 17 polymorphic pairs.

**Table 1:** Computed Relative Lattice Energies (PBE-MBD/B2PLYPD) and Experimental Enthalpy Differences for the Benchmarking Sets of Polymorphs

Validation subset PBE-MBD/B2PLYPD				Test subset PBE-MBD/B2PLYPD			
System	Refcode	Δ*H*_exp_ (kJ/mol)	Δ*E*_latt_ (kJ/mol)	System	Refcode	Δ*H*_exp_ (kJ/mol)	Δ*E*_latt_ (kJ/mol)
o-acetamido benzamide^45^	ACBNZA01	0.0	0.0	anthranilic acid^46^	AMBACO06	0.0	0.0
	ACBNZlA02	2.9	2.5		AMBACO03	1.8	–0.6
cimetidine^47^	CIMETD01	0.0	0.0	4-aminobenzoic acid^48^	AMBNAC04	0.0	0.0
	CIMETD02	2.3	2.6		AMBNAC06	2.8	0.1
probucol^49^	HAXHET	0.0	0.0	benzophenone^50^	BPHENO12	0.0	0.0
	HAXHET01	2.1	1.0		BPHENO11	4.1	2.2
bicalutamide^51,52^	JAYCES	0.0	0.0	4′-hydroxy acetophenone^53^	HACTPH30	0.0	0.0
	JAYCES02	4.7	–3.9		HACTPH31	0.5	4.1
ritonavir^54^	YIGPIO03	0.0	0.0	metacetamol^55^	MENSEE01	0.0	0.0
	YIGPIO02	6.9	6.0		MENSEE04	4.7	5.8
				picolinamide^56^	PICAMD04	0.0	0.0
					PICAMD03	1.1	2.0
				sulfamerazine^57^	SLFNMA01	0.0	0.0
					SLFNMA02	3.5	6.4
				phenylpiracetam^58^	SANBAN	0.0	0.0
					SAMZOY	2.7	–0.7
4-methyl-2-nitroacetanilide^59^	MNIAAN11	0.0	0.0	carbamazepine^60^	CBMZPN10	0.0	0.0
	MNIAAN12	2.4	5.6		CBMZPN11	1.3	3.3
	MNIAAN02	3.4	5.2		CBMZPN12	1.9	–0.3
	**MAD** ^a^		2.3		**MAD** ^a^		2.3

a Stability of 7 pairs of polymorphs for the BLEM test set and 10 pairs of polymorphs for the BLEM validation set.

### 2.4 Example Validation Systems: *o*-Acetamidobenzamide and Ritonavir

In this section, we present crystal energy data for two infamous conformational polymorphic compounds: (a) *o*-acetamidobenzamide (ACBNZA) and (b) ritonavir (YIGPIO). The polymorphism of these two systems has been intensively studied and both compounds are known to crystallize in a metastable form with a lower energy conformer and in the most stable form with a higher-energy conformer.^45,61^ These systems also represent two different archetypes of conformational behaviors that can be challenging to model accurately with electronic structure theory. *O*-acetamidobenzamide has just a few conformational degrees of freedom, but the changes in the conformations between the α and β polymorphs alter the π-conjugation substantially. The density-driven delocalization error inherent in lower-level density functionals artificially stabilizes the more conjugated conformations, leading to incorrect polymorph stability rankings.^14,15,42,43^ In contrast, ritonavir is much larger and is highly flexible. None of the conformations found in the experimental polymorphs of ritonavir differ in their extent of π-conjugation, and their conformational energies are less impacted by delocalization error. Nevertheless, the large molecular size and flexibility of the ritonavir molecule leads to varying intramolecular noncovalent interactions between polymorphs, which can potentially lead to an accumulation of numerous smaller “general” functional errors. Such behaviors have also been observed in molecule XXXII from the seventh Blind Test of Crystal Structure Prediction.^21^ All of these intramolecular behaviors then interplay with the intermolecular interactions in the crystal, complicating the computation of accurate polymorph lattice energy differences. In *o*-acetamidobenzamide, for example, conformational changes convert the intramolecular hydrogen bond in the α form to an intermolecular one in the β polymorph.

These two systems serve, therefore, as excellent test cases for the computational methodologies as well as for illustrating the different intra- versus inter-molecular contributions to the lattice energies as computed with our BLEM (Figure 4). For our discussions, we first focus on the energy models used for the computations and then on the various contributions to the lattice energies of the polymorphs. Only energies for the higher-accuracy intermolecular model (PBE-MBD) are presented.

**Figure 4:**
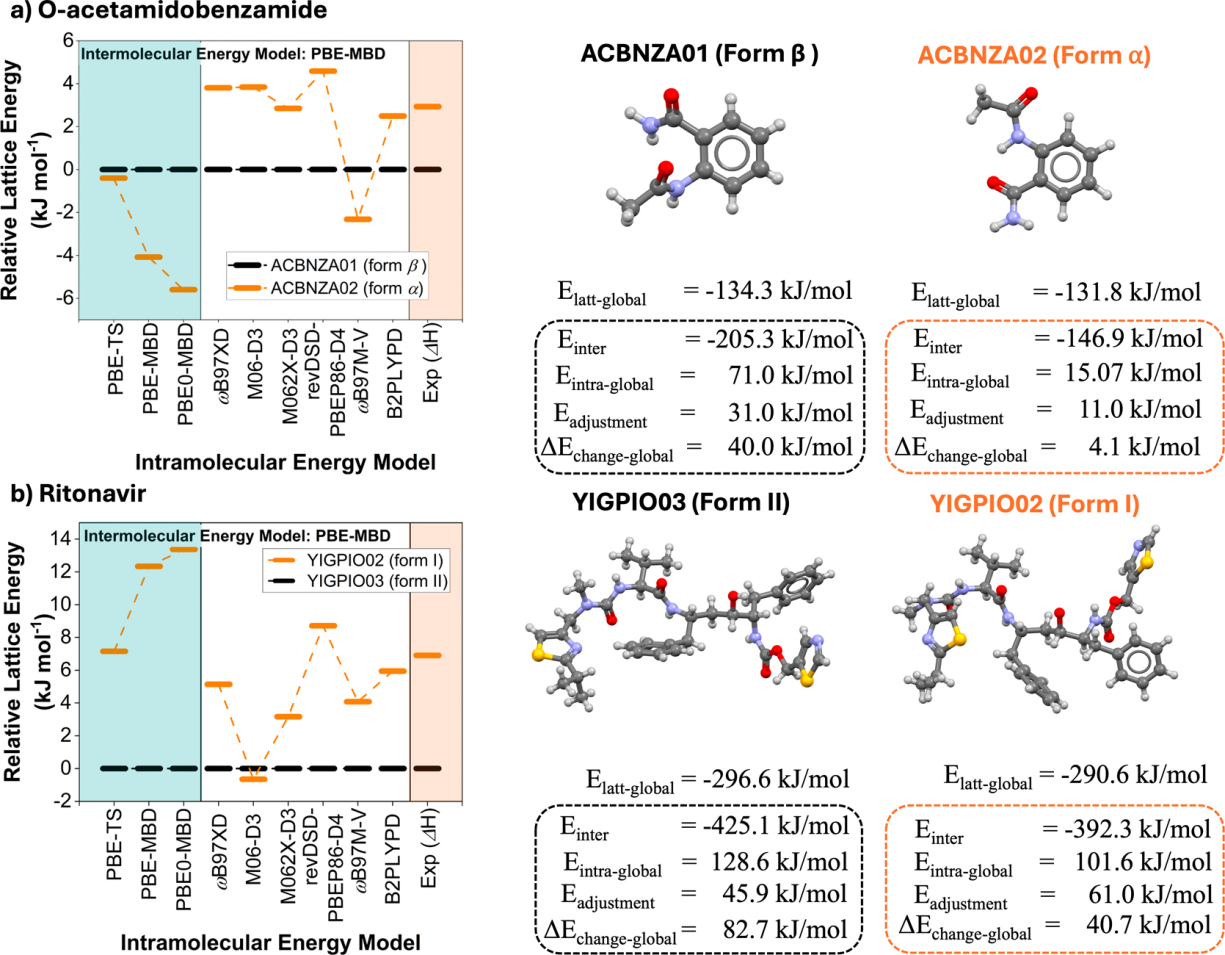
Calculated relative lattice energies (left) of the polymorphs of *o*-acetamidobenzamide and ritonavir as computed with various models. The lattice energies of the polymorphs and their components are also given as computed with the BLEM.

Focusing on *o*-acetamidobenzamide first, we remark that only five of the nine intramolecular energy models are able to reproduce the experimental order of stabilities of the polymorphs. The substantial overstabilization of the α form relative to the β one observed with PBE-TS and PBE-MBD is consistent with the intramolecular delocalization error issues surrounding the conformational energy described above. However, correcting the intramolecular conformational energies with more advanced density functionals usually improves the agreement with experiment. For example, the B2PLYPD model for intramolecular energy combined with the PBE-MBD for intermolecular reproduces the experimental stability difference very accurately: 2.5 kJ/mol computational vs 2.9 kJ/mol experimentally. While the global lattice energies computed for both polymorphs are very similar (∼−132 kJ/mol), their intra- versus inter-molecular contributions are remarkably different. The metastable form α has a small overall *E*_intra-global_ penalty term of just ∼15 kJ/mol, with the major contribution originating from the adjustment energy alone. The conformer in the α form has an intramolecular hydrogen bond, making it very stable. The stable form β, in contrast, has a significantly larger *E*_intra-global_ penalty term of ∼71 kJ/mol. The larger conformational energy penalty is compensated by higher stabilizing intermolecular contributions to the lattice energy (−205.3 vs −146.9 kJ/mol for forms β and α, respectively). This relationship is reflected in experiments, where form α has been reported to crystallize by fast cooling crystallizations from a variety of solvents and the melt with form β obtained from gentler slow cooling conditions or slurry conversions.^45^

Focusing on ritonavir second, while most methods reproduce the order of stabilities, the computed relative stabilities differ significantly, ranging from just 1 kJ/mol to nearly 14 kJ/mol depending on the intramolecular energy method used. The B2PLYPD model for intramolecular energy combined with the PBE-MBD for intermolecular, again, reproduces the experimental stability difference very accurately: 6.0 kJ/mol computational vs 6.9 kJ/mol experimental. The global lattice energies of the ritonavir polymorphs lie between −297 and −291 kJ/mol. Both polymorphs have significant global conformational energy penalties of ∼102 kJ/mol and ∼129 kJ/mol for forms I and II, respectively. The more stabilizing intermolecular energy component in form II compensates for the higher energy conformation observed, lowering the total lattice energy relative to form I. Regarding the experimental crystallization of the system, form II was very difficult to nucleate initially, but once it formed accidentally, form I became a disappearing polymorph as it dissolved to produce the more stable form II.^61^ The two forms can now be reliably produced by crystallization^54^ or milling under different experimental conditions.^62^ Crucially, it was postulated that the higher-energy conformer required for the crystallization of form II may have led to its difficulty of nucleation for the first time.^61^

### 2.5 Data Sets for the Computation of Lattice Energy Partitions

Our goal was to compile a comprehensive and diverse data set of lattice energies using state-of-the-art computational methods to enable detailed analysis of energy partitions. For this, we selected four data sets comprising a total of 125 crystal structures of diverse flexible compounds. The data sets are described in Table 2 and all consist of single-component crystals of neutral organic compounds.

**Table 2:** Datasets Selected for the Computation of Lattice Energies and Their Partitions^a^

Data set	N	$\overset{®}{N\;atoms}$	$\overset{®}{R\;bonds}$	Description
CP	42	47	10	Conformational Polymorphs (CP): this data set contains 42 unique crystal structures of 19 significantly flexible compounds which crystallize as conformational polymorphs.^4,25^
D	17	56	11	Drugs (D): this data set contains 17 crystal structures of highly flexible and large drug compounds.^29^
MCC	32	24	4	Mixed Cruz-Cabeza (MCC): this data set contains a mixture of single-component systems forming 32 unique crystal structures studied at the Cruz-Cabeza’s group in recent years.
MB	34	41	6	Mixed Beran (MB): this data set contains a mixture of single-component systems forming 34 unique crystal structures studied at the Beran’s group in recent years.^43,66–69^
all	125	42	8	Diverse data set of 125 molecular crystals of flexible compounds.

a The table provides the dataset name, the number of crystal structures (N), the average number of atoms ( $\overset{®}{N\;atoms}$ ) and rotatable bonds ( $\overset{®}{R\;bonds}$ ), and a description. All datasets contain single-component neutral compounds.

The first data set, CP (Conformational Polymorphs), includes 42 unique crystal structures from 19 highly flexible compounds which crystallize as conformational polymorphs with significant intramolecular energy differences.^4,25^ The CP data set contains the benchmarking validation subset and is particularly well-suited to our study, as it features mid to large-sized compounds (average of 47 atoms) with substantial flexibility (average of 10 rotatable bonds) and multiple polymorphic forms. The second data set, D (Drugs), comprises 17 unique crystal structures, each corresponding to a distinct large and highly flexible pharmaceutical compound.^29^ These molecules are representative of real-world drug-like systems, with an average of 56 atoms and 11 rotatable bonds. Lattice energies for both the CP and D data sets were computed using the BLEM model (PBE-MBD/B2PLYPD) as part of this study.

The remaining two data sets, MCC and MB, consist of mixed organic single-component systems previously studied by the Cruz-Cabeza and Beran groups. The MCC data set contains the benchmarking test subset. MCC includes 32 crystal structures, and MB includes 34, with compounds that are smaller than the previous two data sets (MCC: 24 atoms, MB: 41 atoms) and moderately flexible (MCC: 4 rotatable bonds, MB: 6 rotatable bonds). Lattice energies for the MCC data set were also computed using the BLEM model. For the MB data set, lattice energies were computed using a separate model (B86bPBE-XDM/SCS-MP2D),^63–65^ as previously reported in prior work. Nevertheless, results from both models should be reasonably comparable, since both employ good-quality dispersion-corrected generalized gradient approximation functionals together with high-quality intramolecular energy corrections. Indeed, the consistency observed among the results computed with these different models emphasizes the general nature of the conformational energy trends identified.

### 2.6 Lattice Energy Partitions and the 40% Limit

In this section, we explore the relationship between the *E*_intra-global_ and *E*_inter_ for the 125 crystal structures across the four data sets. Figure 5a shows that there is a strong relationship between these two energies, where the more negative the *E*_inter_, the higher the intramolecular energy a compound can achieve. This illustrates how higher-energy molecular conformations are stabilized by more favorable intermolecular interactions in the solid-state. The simple relationship *E*_intra-global_ = −0.4­(*E*_inter_) (shown as a red line in Figure 5a) describes the maximum upper bound of all data for the 125 crystal structures of flexible compounds. We refer to this correlation as the 40% limit in lattice energy partitions of molecular crystals. This limit simply states that high-energy conformations in molecular crystals can afford a maximum energy penalty (as computed in the gas-phase) of up to 40% of the intermolecular interactions in the crystal lattice. The intramolecular energy is compensated by the improved intermolecular energy in the crystal, but there exists a limit of up to 40% for such compensation to be realized experimentally. This result is very important since it reveals the high-energy conformations that are physically accessible in the solid-state through compensations in improved intermolecular interactions. Figure 5b presents the distribution of the absolute value of the fraction between the intra- and the inter-molecular contributions to the lattice energies (|*E*_intra-global_/*E*_inter_|). The distribution shows that *E*_intra-global_ is within ∼10% of the |*E*_inter_| for ∼50% of molecular crystals, within ∼15% of the |*E*_inter_| for ∼80% of molecular crystals, and within ∼40% of the |*E*_inter_| in 100% of molecular crystals.

**Figure 5:**
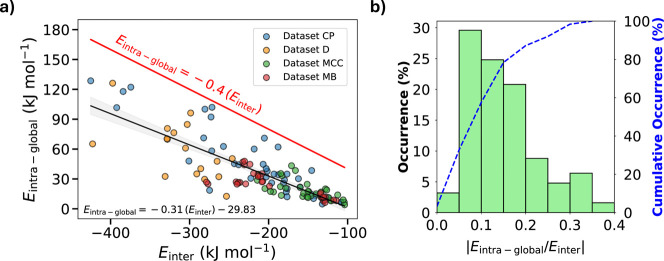
(a) Correlation between *E*_intra-global_ and *E*_inter_ across all 125 crystal structures studied. The black line and equation represent the linear fit to the data, while the red line and equation indicate the upper bound limit. (b) Distribution of the absolute value of the intra-to-intermolecular energy ratio for all data sets. Energies for data sets CP, D, and MCC were calculated using our BLEM approach, whereas MB values were obtained via the comparable B86bPBE-XDM/SCS-MP2D method.

## 3 Discussion

We have shown that the ability of a flexible compound to distort its shape through rotation of bonds is strongly correlated to the number and strength of intermolecular interactions it can form within its crystal lattice. Our quantification of this phenomenon shows that about 50% of molecules incur conformational energy penalties equivalent to 10% of the magnitude of the lattice intermolecular interaction energies |*E*_inter_|. The likelihood of higher-energy conformations being observed experimentally decreases as a function of intra-to-intermolecular energy ratio, |*E*_intra-global_/*E*_inter_|, becoming negligible at 0.4. This trend offers valuable insights into molecular self-assembly and has significant implications for crystal engineering and experimental crystallization. To our knowledge, intramolecular energies in molecular crystals have previously only been reported in absolute terms;^4,24^ expressing them as a fraction of the intermolecular lattice energies (|*E*_intra-global_/*E*_inter_|) provides a more robust framework for comparing energy distributions across crystals of molecules of varying complexities, sizes, and flexibilities.

Crystal formation involves both nucleation and growth. For flexible compounds, adopting the appropriate crystal conformation is a prerequisite for incorporation into a growing crystal.^30–34^ Crystal growth occurs either at flat surfaces, step edges, or kinks—with the kink incorporation being the dominant mechanism at low supersaturations. Notably, at a kink site (also known as the half-crystal position as defined by Kossel and Stranski),^70,71^ a molecule engages with approximately half of its potential lattice neighbors, utilizing ∼50% of the total lattice intermolecular interactions during crystal growth.^72–74^ A conformation with intramolecular energy of 40%|*E*_inter_| can still gain a stabilization of 10%|*E*_inter_| upon incorporation into a kink site. Incorporating conformations with even higher intramolecular energy penalties (>50%|*E*_inter_|) would be energetically unfavorable, as they would not benefit from a net energy gain during kink incorporation—thus impeding crystal growth. We propose, therefore, that our 40% limit is a consequence of the energetics of crystal growth of flexible compounds. Growth at step edges and flat surfaces involve fewer intermolecular interactions, therefore only permitting the incorporation of conformations with lower intramolecular energy. The overall distribution of |*E*_intra-global_/*E*_inter_| may reflect the energetics involved in the mechanism of crystal growth.

Our 40% limit and the associated probability distributions of |*E*_intra-global_/*E*_inter_| offer valuable tools for crystal engineering, particularly in guiding crystal design and prediction. For instance, our findings indicate that a comprehensive exploration of CSP landscapes must include conformations with intramolecular energies up to 40% of |*E*_inter_|. This threshold ensures that all conformations capable of physically packing into the solid-state are considered. This energy window, however, can be substantial for large and highly flexible molecules such as ritonavir with an intramolecular energy threshold of ∼170 kJ/mol (see Figure 4). Despite the magnitude of these values for some systems, including conformations within the 40% limit is essential for achieving completeness and accuracy in CSP landscapes. This challenge is increasingly recognized, and databases cataloguing conformational landscapes of complex molecules are now being developed to support the community and to fit faster ML energy models for such explorations.^75^

In terms of interpreting CSP landscapes, our probability distribution of |*E*_intra-global_/*E*_inter_| (Figure 5b) can be used to inform “crystallizability” of flexible molecules. Plotting CSP landscapes against this ratio would provide a meaningful way to incorporate the likelihood of distorted conformations being experimentally realized through crystallization, thereby bridging computational predictions with experimental outcomes. Furthermore, a decomposition of the intramolecular energy term *E*_intra-global_ into its constituent components—adjustment and change (Figure 6)—revealed that conformational distortion is predominantly driven by adjustment (Figure 6a,b). As a result, both *E*_intra-global_ and its *E*_adjustment_ component can serve as effective indicators of crystallizability within CSP landscapes.

**Figure 6:**
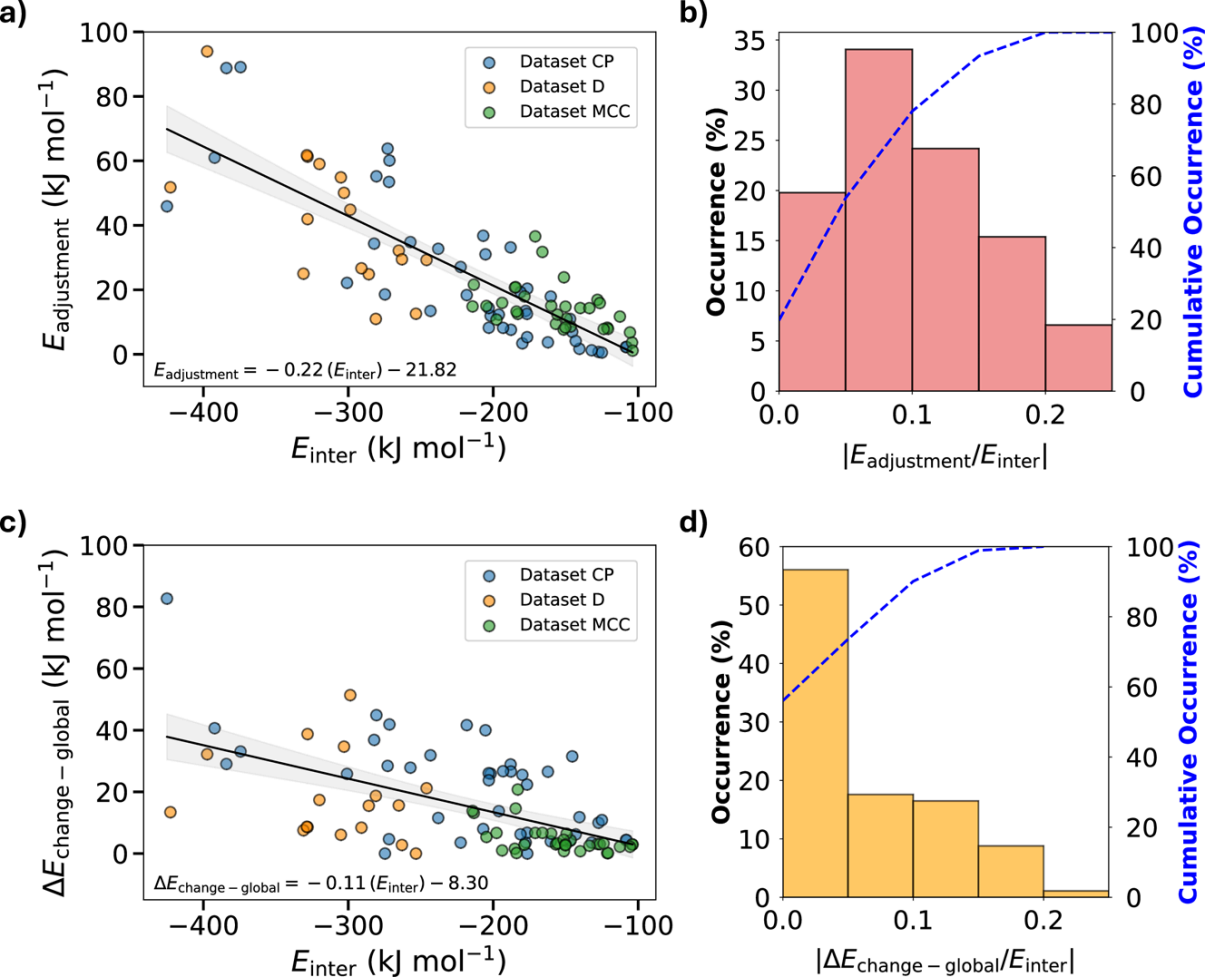
(a) Correlation between *E*_adjustment_ and *E*_inter_ across all 125 crystal structures studied. The black line and equation represent the linear fit to the data. (b) Distribution of intra­(adjustment)-to-intermolecular energy ratio for all data sets. (d) Correlation between Δ*E*_change‑global_ and *E*_inter_ across all 125 crystal structures studied. The black line and equation represent the linear fit to the data. (b) Distribution of intra­(change)-to-intermolecular energy ratio for all data sets. Energies for data sets CP, D, and MCC were calculated using our BLEM approach, whereas MB values were obtained via the comparable B86bPBE-XDM/SCS-MP2D method.

Conformational polymorphs exemplify how conformational energies may impact crystallizability. Revisiting the specific cases discussed in Section 2.4, we note that both stable conformational polymorphs of *o*-acetamidobenzamide (form β) and ritonavir (form II) have consistently been reported as difficult to crystallize. In both systems, the absolute intra-to-inter fractions are notably high, with values of 0.35 for *o*-acetamidobenzamide form β and 0.30 for ritonavir form II. For *o*-acetamidobenzamide, early work by Errede et al.^45^ indicated that form α is significantly more prevalent under most crystallization conditions and easier to crystallize than form β. Despite attempts in our laboratory to produce form β *o*-acetamidobenzamide using the conditions reported by Errede et al.,^45^ we were unsuccessful and instead obtained a cocrystal with a degradation product.^76^ Leech et al. succeeded in crystallizing form β only after preparing partially deuterated polycrystalline material.^77^ The case of ritonavir form II is very well-known; it emerged as a late-appearing polymorph and was notoriously difficult to nucleate initially.^61^ The similarity in the difficulty of crystallization of these two polymorphs is striking. Despite both being stable polymorphs, their nucleation is hindered—an observation that correlates with their high intra-to-inter energy fractions. These examples further underscore the utility of lattice energy partitioning as a predictive tool for assessing “crystallizability”.

Finally, it is important to note that the data presented here pertains to crystals of single-component neutral compounds. While we anticipate similar trends may apply to multicomponent crystals composed of neutral species, systems involving charged components require separate consideration. In these systems, charge transfer notably amplifies the electrostatic components of intermolecular interactions within the crystal, making it challenging to quantify their influence on crystal conformations. For ions and zwitterions, evaluating intramolecular energies in the gas phase may be unsuitable, as such conditions strongly disfavor their stability. These systems will be investigated further in future studies.

## 4 Conclusions

After identifying an excellent computational model (PBE-MBD/B2PLYPD) to reproduce lattice energies of crystals of flexible molecules (and their partitions), we have conducted a detailed analysis of intra- and inter- molecular contributions to lattice energies of crystals across a diverse data set of 125 structures. This investigation led to the formulation of the 40% limit of lattice energy partitioning, which establishes that flexible molecules can tolerate intramolecular energy penalties up to 40% of their intermolecular stabilization energy in the solid-state. Moreover, the probability of observing high-energy conformations decreases linearly from a peak at 10% of the intrato-inter energy fraction, becoming negligible at the 40% threshold. Further decomposition of intramolecular energy into adjustment and change components revealed that conformational distortion is primarily driven by adjustment.

The 40% limit offers a practical upper bound for conformational distortion in experimentally observed crystal structures, while the intrato-inter energy fraction emerges as a predictive metric for crystallizability. While an empirical observation, the “40% limit” has the potential to evolve into a rule in the future as more evidence is gathered beyond the 125 crystal structures studied here. We propose that these observations are rooted in crystal growth mechanisms, particularly kink incorporation, where molecules engage only half of their potential lattice interactions—thus defining the energetic limit for the inclusion of high-energy conformations. Taken together, these insights establish a quantitative foundation for understanding the energetic constraints of molecular flexibility in the solid-state, offering a predictive tool to crystallization outcomes.

## 5 Methods

### 5.1 Data Sets, Methods and Structural Models

The CP, D and MCC data sets were all treated, and their energies computed with the methods described in this section. Crystal structure data for the CP, D and MCC data sets were retrieved from the CSD using Conquest.

The MB data set and its energies (B86bPBE-XDM/SCS-MP2D) were taken from prior work.^43,66–69^ Briefly, for the intermolecular energy calculations, periodic B86bPBE-XDM was used with a 50 Ry PlaneWave cutoff and Monkhorst–Pack *k*-point spacing of at least 0.05 Ang^–1^ in QuantumEspresso.^78^ The intramolecular energies were subsequently corrected with SCS-MP2D energies computed using PSI4 ^79^ and the MP2D library.^80^ They were extrapolated to the complete basis set limit from the aug-cc-pVTZ and aug-cc-pVQZ basis sets.

Conformational searches were performed for all systems from all databases as described below.

#### 5.1.1 Energy Partition

Global lattice energies (*E*_latt‑global_) were calculated by adding the independently computed intermolecular (*E*_inter_) and intramolecular (*E*_intra-global_) energy components (eq 2). Intramolecular energies are calculated by adding the computed adjustment and change energy terms (eq 3).

2 $$E_{latt‐global}=E_{inter}+E_{intra‐global}$$

3 $$E_{intra‐global}=E_{adjustment}+\Delta E_{change‐global}$$

#### 5.1.2 Computation of Intermolecular Energies

Intermolecular energies were computed using the Vienna Ab initio Simulation Package (VASP 6.3.2) code^81–83^ and the PBE functional.^84^ The electronic calculations were carried out using plane waves with a 520 eV energy cutoff, the Brillouin Zone sampled using Monkhorst–Pack *k*-point meshes^85^ with grid spacing of at least 0.03 Å^–1^. Two energy models were used for the computation of the final intermolecular interactions: (a) the PBE functional with Tkatchenko–Scheffler dispersion corrections (PBE-TS)^86^ and (b) the PBE-TS model with the addition of many body dispersion corrections (PBE-MBD).^18,19^ These two energy models were used to calculate the energy of the 42 crystal structures after an extensive geometry optimization procedure allowing all unit cell parameters to relax.

The following computational procedure was used. First, the structures retrieved from the CSD were geometry optimized twice using the PBE functional with Grimme D2 dispersion corrections^87^ and allowing all unit cell parameters to relax. Second, two further geometry optimizations of the crystal structures were carried out at the PBE-TS level of theory. Crystal geometry outputs from prior optimizations were used as starting inputs for the next ones. Geometry optimization convergence was achieved when the residual force per atom was less than 10^–5^ eV Å^–1^. Third, two single point energy calculations were then performed at PBE-TS and PBE-MBD levels of theory. Energy outputs from this final step (*E*_crystal_^VASP‑Opt/*SP*^) were used for the calculation of intermolecular energies. For this, the energies obtained from VASP in eV per simulation cell (which in this case equals the unit cell) were normalized by the number of molecules in the simulation cell leading to the computation of the electronic energy per molecule in the crystal (*E*_emol‑crystal_^VASP‑Opt/SP^). Taken the fully relaxed crystal, a single crystal conformation was extracted and taken for further simulations. We will refer to this molecular geometry as the crystal conformation.

The crystal conformation was placed in isolation in a large cubic supercell of 30 × 30 × 30 Å^3^ dimensions. Simulation of the crystal conformation in this large supercell provides a reasonable approach for modeling the conformation in the gas-phase. Two single point energy calculations were carried out for the supercell containing the crystal conformation at the two levels of theory used (*E*_emol‑gas_^VASP‑SP^). The intermolecular energy of the crystal *E*_inter_ was then calculated by subtracting the electronic energy of the crystal conformation in the gas-phase (*E*_emol‑gas_^VASP‑SP^) from the electronic energy of the crystal conformation in the crystal (*E*_emol‑crystal_^VASP‑Opt/SP^), eq 4. Two sets of intermolecular energies were calculated, one with the PBE-TS model and a second one with the PBE-MBD.

4 $$E_{inter}=E_{emol‐crystal}^{VASP‐Opt/SP}-E_{emol‐gas}^{VASP‐SP}$$

#### 5.1.3 Procedure for the Computation of Adjustment Energies

The adjustment energy is the component of the intramolecular energy related to the energy penalty paid when placing a gas-phase conformer into a crystal as the conformation adjusts to the intermolecular forces in the solid-state. It is calculated as the difference between the electronic energy of the crystal conformation in the gas-phase minus the electronic energy of its corresponding conformer in the gas-phase after relaxing to the nearest energetic local minimum. To obtain the energy of the crystal conformation in the gas-phase, a simple single point (SP) energy calculation of the geometry extracted from the optimized crystal and placed in isolation is carried out (*E*_emol‑gas_^SP^) at the desired level of theory. To obtain the energy of the gas-phase conformer for a given crystal conformation, the crystal conformation is fully geometry optimized (Opt) in the gas-phase and its energy computed (*E*_emol‑gas_^opt^). The adjustment energy is then calculated as in eq 5. Adjustment energies are always positive since they are an energy penalty illustrating the cost of the conformation adapting to the crystal field.

5 $$E_{adjustment}=E_{emol‐gas}^{SP}-E_{emol‐gas}^{Opt}$$

#### 5.1.4 Procedure for the Computation of Change Energies

The change energy is the component of the intramolecular energy related to the energy required to generate the current gas-phase conformer starting from another gas-phase conformer. The change energy can be calculated relative to different reference conformers, such as relative to the conformation found in another polymorph or the global minimum energy conformation. Here we calculate the global change energy for the crystal under study relative to the most stable conformer generated computationally from a conformer generation search (*E*_emol‑gas_^Opt‑GenMin^) as shown in eq 6.

6 $$\Delta E_{change‐global}=E_{emol‐gas}^{Opt}-E_{emol‐gas}^{Opt‐Genmin}$$

#### 5.1.5 Global Conformer Search

For all 125 systems studied, a conformational search was conducted to identify the global energy conformer per compound. This was done using the Conformer Generation tool within the CSD-Materials module available in Mercury.^88^ Briefly, for each compound, Conformer Generation searches were performed starting from each observed crystal conformation. A maximum of 10^6^ conformations were sampled per search using the standard search parameters in the tool such as a maximum of two unusual torsions allowed in each of the generated conformations. A maximum of 200 conformations were produced from which the top five most likely conformers (according to the CSD probabilities) were then taken for further optimization with the M06-D3 method^89^ followed by a single point energy calculation with the double hybrid B2PLYPD model.^90^ The M06-D3 geometry optimizations and B2PLYPD single point energy calculations used a simpler split valence basis set, 6-31+G­(d,p), to account for computational tractability.^91^ The most stable conformer per system were then further optimized with the B2PLYPD single point energy calculations using a triple-ζ valence basis set with double polarization (def2-TZVPP) for the computation of Δ*E*_change‑global_.^92^ For the MB data set, the stable conformer was computed with the Δ­(SCS-MP2D) for the computation of Δ*E*_change‑global_ with the model consistent with the previous data.

#### 5.1.6 Gas-Phase Electronic Energy Methods for the Computation of Intramolecular Energies

The intramolecular energies for all conformations were calculated using several models as implemented in three different electronic energy software. First, the supercell approach (as explained above) was used for the computation of intramolecular energies at the PBE-TS and the PBE-MBD levels of theory in VASP. The PBE0-MBD model was also used, but only with light settings due to the expense of the computations. The supercell is needed since VASP operates with periodic boundary conditions and planewaves. For the geometry optimizations in the supercell models, the supercell vectors were kept fixed and only the atomic positions of the conformations optimized. Computational parameters used are identical to those applied for the computation of intermolecular energies as presented above, aside from unit cells which were always kept fixed. Second, the localized basis-set codes Gaussian16 and ORCA were also used to explore several other methods for the computational energies. Within ORCA, the following methods were investigated: revDSD-PBEP86-D4 and ωB97M-V.^93^ Within Gaussian16, the following methods were investigated: M06-D3, M062X-D3,^89^ ωB97XD^94^ and B2PLYPD. All geometry optimizations and single point energy calculations were performed using the def2-QZVPP basis set, except for the B2PLYPD calculations which were performed using the def2-TZVPP basis set due to the expense of the computations.^92,95^

## Supplementary Material



## Notes

The Supporting Information contains crystal energies and computations of inter- and intramolecular energies, and energy distributions for all test systems using all nine intramolecular energy models (PDF)
