# Supplementary material for: Lattice Energy Partitions in Crystals of Flexible Molecules and the 40% Limit
Source: J Am Chem Soc. 2025 Oct 15;147(43):39192–203. doi: 10.1021/jacs.5c09997 (PMC12576777; doi:10.1021/jacs.5c09997)
Supplement: Supplementary file 1 [file ja5c09997_si_001.pdf]

Supplementary Material

Lattice Energy Partitions in Crystals of Flexible Molecules and the 40% Limit

Amrita Chattopadhyay,[a] Adam R. Hill, [a,b] Sarah E. Wright,[c] Gregory J. O. Beran[d] and Aurora J. Cruz-Cabeza [a*]

[a] Department of Chemistry, Durham University, Durham DH1 3LE, United Kingdom.

[b] Department of Chemistry, The University of Manchester, Manchester M139PL, United Kingdom.

[c] Department of Chemical Engineering, The University of Manchester, Manchester M139PL, United Kingdom.

[d] Department of Chemistry, University of California Riverside, Riverside, California 92521, United States.

*Corresponding author, email: aurora.j.cruz-cabeza@durham.ac.uk

Table of Contents

1. Selection and Testing of BLEM ***2***

2. Generation and Prediction of Global Minima Conformers ***2***

3. Lattice Energy Partitions ***2***

3.1. Dataset CP (conformational polymorphs) **4**

3.2. Dataset D (drugs) **6**

3.3. Dataset MCC (mixed cruz-cabeza) **7**

3.4. Dataset MB (mixed beran) **8**

4. Supplementary References ***10***

1. The BLEM and the Validation Subset

The performances of all intramolecular methods[1,2–8] for in combination with the PBE-MBD intermolecular method for all validation systems are presented in Figure S1.

2. Generation and Prediction of Global Minima Conformers

A search was conducted to identify the global conformer for each polymorph form. The Conformer Generation tool within the CSD-Materials available in the CSD Python API was used to generate up to 200 conformers from which, the five conformers with highest probability were selected for each compound.[9] The selected conformers were minimised in Gaussian using dispersion corrected M06-D3 method[10] followed by a single point energy calculation with the double hybrid model B2PLYPD.[11] All calculations were carried out using a simpler split valence basis set – 6-31+G(d,p)[12] – with a double polarization function to account for computational tractability. After identifying the most stable global conformers per system, their energies were recomputed using a single point energy calculation with B2PLYPD/Def2TZVPP.[13] We note that conformers for Dataset MB were optimised and their energies calculated slightly differently. For this, the M06-D3 method was used for optimisation followed by the Δ(SCS-MP2D) method for the final calculation of intramolecular energies.[14]

3. Lattice Energy Partitions

The partition of lattice energies for the CP, D and MCC datasets are presented in tables S1-3 for data obtained with the BLEM. Additionally, information for the MB dataset is presented in table S4 for the MB dataset as computed with the B86bPBE-XDM/Δ(SCS-MP2D) method. All tables also provide molecular descriptors such as the number of atoms per molecule (MolAtoms), number of rotatable bonds per molecule (RotatableBonds), and the rotatable bonds normalised against heavy atoms (RotatableBondsPer100HeavyAtoms) in %.

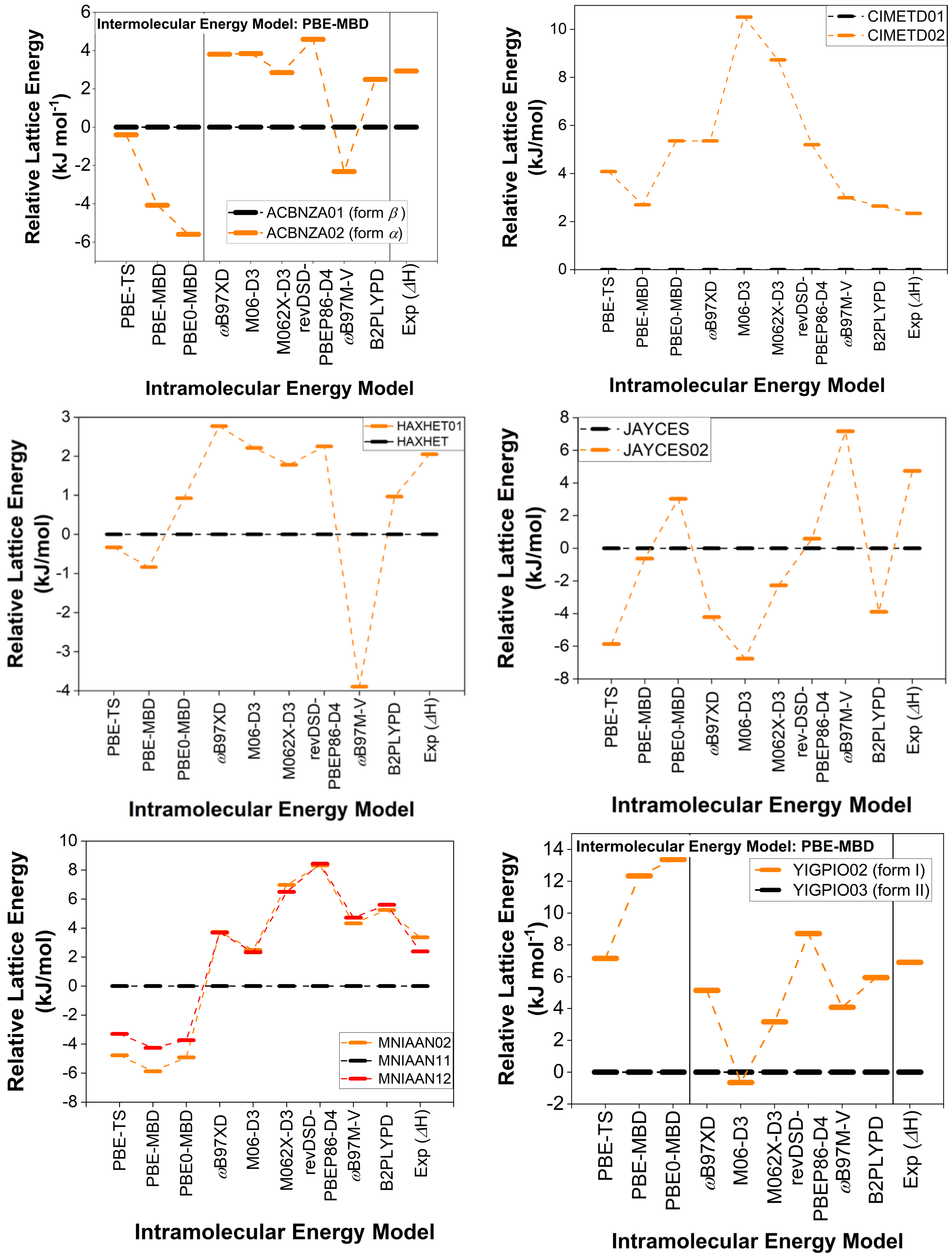

Figure S 1 Performance of all intramolecular energy models against the relative lattice energies of the validation subset. Lattice energies were calculated using the PBE-MBD intermolecular energy model. All energies are in units of kJ/mol.

3.1. Dataset CP (conformational polymorphs)

Table S 1 Inter- and intramolecular energies and structural data of crystal structures of conformational polymorphs.

Compound	Mol Atoms	*R*-Bonds	*R*-Bonds Per100Heavy Atoms (%)	E_{inter} (kJ/mol)	$E_{intra-adjustment}$ (kJ/mol)	$\Delta E_{change-global}$ (kJ/mol)	$E_{intra-global}$ (kJ/mol)	$E_{latt-global}$ (kJ/mol)	$\lvert E_{intra-global}/E_{inter}\rvert$ ratio
ACBNZA01	23	5	38	-205.34	31.01	39.99	71.00	-134.34	0.35
ACBNZA02	23	5	38	-146.92	10.95	4.12	15.07	-131.85	0.10
CELBEA02	35	9	50	-201.77	11.93	25.92	37.86	-163.91	0.19
CELBEA	35	9	50	-203.14	14.35	26.06	40.41	-162.73	0.20
CELBEA01	35	9	50	-177.59	13.45	3.61	17.06	-160.53	0.10
CIMETD01	33	10	59	-257.14	34.80	27.85	62.65	-194.49	0.24
CIMETD02	33	10	59	-222.47	27.08	3.54	30.62	-191.85	0.14
EYOCUQ01	45	12	52	-384.12	88.80	29.05	117.85	-266.27	0.31
EYOCUQ	45	12	52	-374.37	89.08	33.10	122.19	-252.19	0.33
GLUCIT02	26	11	92	-272.86	63.79	28.43	92.22	-180.64	0.34
GLUCIT04	26	11	92	-280.60	55.21	44.89	100.10	-180.49	0.36
GLUCIT03	26	11	92	-271.61	60.11	41.87	101.98	-169.63	0.38
HAXHET	83	24	69	-203.04	8.23	23.77	32.00	-171.04	0.16
HAXHET01	83	24	69	-196.13	12.30	13.76	26.06	-170.07	0.13
ICIMAI	30	8	36	-176.50	20.36	0.00	20.36	-156.14	0.12
ICIMAI01	30	8	36	-179.89	3.39	25.53	28.92	-150.97	0.16
JAYCES02	43	10	34	-206.89	36.77	7.97	44.74	-162.15	0.22
JAYCES	43	10	34	-218.28	18.36	41.66	60.03	-158.25	0.27
KIJBOX	42	7	25	-282.17	34.33	36.85	71.18	-210.98	0.25
MCHTEP11	52	16	44	-188.00	33.16	28.90	62.06	-125.93	0.33
MCHTEP13	52	16	44	-127.34	0.73	9.98	9.25	-118.09	0.07
MCHTEP07	52	16	44	-125.00	0.54	10.86	11.40	-113.60	0.09

MELVEE	59	7	23	-176.61	5.31	6.75	12.05	-164.56	0.07
MELVEE01	59	7	23	-187.78	7.60	26.61	34.21	-153.57	0.18
MIQKOM	42	0	0	-193.21	8.20	26.70	34.89	-158.32	0.18
MIQKOM01	42	0	0	-181.15	19.28	6.24	25.51	-155.64	0.14
MNIAAN11	24	5	36	-145.47	6.95	31.52	38.47	-107.00	0.26
MNIAAN02	24	5	36	-108.41	2.25	4.41	6.66	-101.75	0.06
MNIAAN12	24	5	36	-108.00	2.24	4.37	6.61	-101.39	0.06
QIJZOY	42	8	29	-271.93	53.47	4.67	58.14	-213.80	0.21
TOHBUN01	68	14	41	-274.71	18.57	0.00	18.57	-256.13	0.07
TOHBUN02	68	14	41	-300.90	22.15	25.84	47.99	-252.91	0.16
TTHCTD	68	0	0	-132.08	1.24	3.56	4.80	-127.28	0.04
TTHCTD01	68	0	0	-140.50	1.69	11.79	13.48	-127.02	0.10
UFAGIS01	26	5	25	-143.08	4.12	6.21	10.33	-132.75	0.07
UFAGIS	52	10	25	-162.27	3.71	26.54	30.25	-132.02	0.19
WUCGUW02	63	8	28	-243.30	13.45	31.87	45.32	-197.98	0.19
WUCGUW	63	8	28	-237.95	32.72	11.54	44.26	-193.69	0.19
YIGPIO03	98	28	56	-425.14	45.89	82.69	128.58	-296.55	0.30
YIGPIO02	98	28	56	-392.25	60.99	40.65	101.64	-290.61	0.26
ZEYBIO04	56	8	25	-176.69	12.53	22.37	34.89	-141.80	0.20
ZEYBIO01	28	4	25	-160.28	17.91	3.94	21.86	-138.43	0.14
**Average value**	**47**	**10**	**40**	**-215.78**	**23.99**	**21.09**	**45.08**	**-170.70**	**0.19**

3.2. Dataset D (drugs)

Table S 2 Inter- and intramolecular energies and structural data of crystal structures of single component drug compounds.

Compound	Mol Atoms	*R*-Bonds	*R*-Bonds Per100Heavy Atoms (%)	E_{inter} (kJ/mol)	$E_{intra\text{-}adjustment}$ (kJ/mol)	$\Delta E_{change\text{-}global}$ (kJ/mol)	$E_{intra\text{-}global}$ (kJ/mol)	$E_{latt\text{-}global}$ (kJ/mol)	$\lvert E_{intra\text{-}global}/E_{inter}\rvert$ ratio
BEWKUM01	35	5	25	-302.97	50.08	34.67	84.75	-218.23	0.28
DIGOXN10	119	18	33	-422.74	51.80	13.41	65.20	-357.54	0.15
KIPLIG	61	15	47	-305.13	54.88	6.09	60.97	-244.16	0.20
MECWIC04	27	6	38	-265.16	32.15	15.61	47.75	-217.41	0.18
MELKIA	45	13	57	-328.12	61.30	8.55	69.85	-258.27	0.21
MOVYAY	77	11	31	-285.87	24.83	15.50	40.33	-245.54	0.14
NIQBIB	58	10	32	-319.94	59.03	17.38	76.42	-243.52	0.24
ODEROH06	51	8	24	-262.99	29.46	2.77	32.23	-230.76	0.12
OVAYOB	66	12	30	-330.94	25.00	7.54	32.54	-298.40	0.10
RSERPN01	84	16	36	-281.01	10.97	18.70	29.67	-251.33	0.11
SAXFED01	58	11	35	-290.93	26.67	8.40	35.07	-255.86	0.12
SUCROS43	45	13	57	-328.58	61.74	8.68	70.42	-258.16	0.21
UGIVAI01	31	8	44	-328.11	41.93	38.75	80.68	-247.44	0.25
USOCOV	70	12	32	-253.32	12.57	0.00	12.57	-240.75	0.05
VEMRAI	52	12	38	-246.00	29.26	21.18	50.44	-195.56	0.21
VIRAZL01	29	7	41	-298.64	44.85	51.38	96.24	-202.40	0.32
YADSOL	47	17	74	-397.39	93.96	32.21	126.17	-271.22	0.32
**Average value**	**56**	**11**	**40**	**-308.70**	**41.79**	**17.70**	**59.49**	**-249.21**	**0.19**

3.3. Dataset MCC (mixed cruz-cabeza)

Table S3 Inter- and intramolecular energies and structural data of crystal structures of single component structures from Cruz-Cabeza group research.

Compound	Mol Atoms	*R*-Bonds	*R*-Bonds Per100Heavy Atoms (%)	E_{inter} (kJ/mol)	$E_{intra\text{-}adjustment}$ (kJ/mol)	$\Delta E_{change\text{-}global}$ (kJ/mol)	$E_{intra\text{-}global}$ (kJ/mol)	$E_{latt\text{-}global}$ (kJ/mol)	$\|E_{intra\text{-}global}/E_{inter}\|$ ratio
AMBACO03	17	3	30	-128.06	16.89	3.10	19.99	-108.07	0.16
AMBACO06	17	3	30	-126.45	15.90	3.05	18.96	-107.50	0.15
AMBNAC04	17	3	30	-139.93	14.34	0.75	15.09	-124.83	0.11
AMBNAC06	17	3	30	-151.21	23.87	0.77	24.64	-126.57	0.16
BAPLOT03	21	2	15	-146.53	8.59	4.32	12.92	-133.61	0.09
BAPLOT06	21	2	15	-149.39	11.05	6.44	17.50	-131.89	0.12
BEDMIG10	30	7	41	-185.08	20.69	1.56	22.25	-162.83	0.12
BEDMIG21	30	7	41	-178.41	17.95	2.88	20.83	-157.58	0.12
BPHENO11	24	2	14	-104.20	3.65	2.95	6.60	-97.60	0.06
BPHENO12	24	2	14	-103.82	1.12	2.91	4.03	-99.79	0.04
BZAMID05	16	2	22	-121.34	8.25	0.16	8.41	-112.92	0.07
BZAMID08	16	2	22	-120.80	7.87	0.15	8.03	-112.77	0.07
CBMZPN10	30	2	11	-156.77	9.44	2.97	12.40	-144.36	0.08
CBMZPN11	30	2	11	-155.69	12.31	3.35	15.66	-140.04	0.10
CBMZPN12	30	2	11	-151.41	7.67	4.47	12.13	-139.27	0.08
CBMZPN16	30	2	11	-150.19	8.32	2.93	11.26	-138.93	0.07
HACTPH30	18	3	30	-133.64	14.32	2.53	16.85	-116.80	0.13
HACTPH31	18	3	30	-123.78	7.83	3.27	11.10	-112.68	0.09
MENSEE01	20	4	36	-159.98	15.03	6.48	21.51	-138.47	0.13
MENSEE04	20	4	36	-150.14	14.78	2.69	17.47	-132.67	0.12
PICAMD03	15	2	22	-105.63	6.78	2.20	8.98	-96.65	0.09

PICAMD04	15	2	22	-112.58	11.70	2.22	13.92	-98.66	0.12
SAMZOY	30	4	25	-183.06	12.52	20.77	33.29	-149.77	0.18
SANBAN	30	4	25	-184.51	20.82	14.61	35.42	-149.08	0.19
SLFNMA01	30	5	28	-193.83	15.95	1.01	16.97	-176.86	0.09
SLFNMA02	30	5	28	-183.93	13.26	0.18	13.44	-170.48	0.07
SUCACB07	28	10	63	-171.01	36.58	6.70	43.28	-127.73	0.25
SUCACB11	28	10	63	-166.06	31.75	6.72	38.47	-127.59	0.23
TUXPEJ	35	6	27	-204.69	14.99	5.37	20.36	-184.34	0.10
YULGIW	35	6	27	-197.72	10.76	6.69	17.45	-180.27	0.09
TeDi_I	24	4	29	-213.37	21.60	13.18	34.78	-178.59	0.16
TeDi_II	24	4	29	-214.26	14.84	13.84	28.68	-185.58	0.13
**Average value**	**24**	**34**	**27**	**-155.23**	**14.11**	**4.73**	**18.83**	**-136.40**	**0.12**

3.4. Dataset MB (mixed beran)

Table S4 Inter- and intramolecular energies and structural data of crystal structures of Beran research group.

**Compounds**	**Mol Atoms**	***R*-Bonds**	***R*-Bonds Per100Heavy Atoms (%)**	**E_{inter} (kJ/mol)**	**$E_{intra\text{-}global\ (SCS\text{-}MP2D)}$ (kJ/mol)**	**E_{latt} (kJ/mol)**	**$\lvert E_{intra\text{-}global}/E_{inter}\rvert$ ratio**
VUSDIX	46	7	25	-241.1	27.0	-214.0	0.11
VUSDIX01	46	7	25	-236.3	26.5	-209.8	0.11
VUSDIX03	46	7	25	-238.4	26.2	-212.3	0.11
VUSDIX04	46	7	25	-240.0	24.8	-215.2	0.10
VUSDIX06	46	7	25	-229.0	25.0	-204.1	0.11
GICKEM	55	10	29	-223.6	44.7	-179.0	0.20
GICKEM01	55	10	29	-232.6	47.0	-185.6	0.20
GICKEM02	55	10	29	-230.8	44.2	-186.6	0.19

DORDUM	47	5	18	-208.2	35.3	-172.9	0.17
DORDUM01	47	5	18	-198.9	22.8	-176.1	0.11
DORDUM02	47	5	18	-213.3	34.2	-179.1	0.16
DORDUM03	47	5	18	-207.4	27.5	-179.9	0.13
DORDUM04	47	5	18	-210.5	32.2	-178.4	0.15
DORDUM05	47	5	18	-213.5	32.6	-181.0	0.15
DORDUM06	47	5	18	-219.0	35.6	-183.4	0.16
DORDUM07	47	5	18	-208.4	27.1	-181.3	0.13
DORDUM10	47	5	18	-230.5	47.8	-182.6	0.21
QAXMEH	27	5	28	-131.3	11.4	-119.9	0.09
QAXMEH01	27	5	28	-131.0	8.1	-122.9	0.06
QAXMEH02	27	5	28	-133.6	11.5	-122.1	0.09
QAXMEH03	27	5	28	-132.5	11.2	-121.3	0.08
QAXMEH04	27	5	28	-129.2	8.7	-120.6	0.07
QAXMEH05	27	5	28	-130.0	11.4	-118.6	0.09
QAXMEH12	27	5	28	-128.6	6.3	-122.2	0.05
QAXMEH52	27	5	28	-131.2	11.2	-120.1	0.09
QAXMEH53	27	5	28	-132.3	11.8	-120.5	0.09
QAXMEH31	27	5	28	-134.8	14.5	-120.3	0.11
QAXMEH57	27	5	28	-134.0	15.9	-118.0	0.12
QAXMEH60	27	5	28	-128.3	12.0	-116.3	0.09
QAXMEH90	27	5	28	-130.5	12.0	-118.5	0.09
ZEHFUR	32	5	25	-114.26	8.12	-106.14	0.07
ZEHFUR02	32	5	25	-117.71	8.81	-108.90	0.07
JEKVII	76	12	27	-276.72	24.97	-251.75	0.09
JEKVII01	76	12	27	-278.65	26.82	-251.82	0.10
**Average value**	**41**	**6**	**25**	**-184.59**	**22.80**	**-161.79**	**0.12**

4. References

1 G. J. O. Beran, S. E. Wright, C. Greenwell and A. J. Cruz-Cabeza, *J Chem Phys*, 2022, **156**, 104112.

2 L. A. Errede, M. C. Etter, R. C. Williams and S. M. Darnauer, *Journal of the Chemical Society, Perkin Transactions 2*, 1981, 233–238.

3 A. Bauer-Brandl, *Int J Pharm*, 1996, **140**, 195–206.

4 J. J. Gerber, M. R. Caira and A. P. Lötter, *Journal of Crystallographic and Spectroscopic Research*, 1993, **23**, 863–869.

5 D. R. Vega, G. Polla, A. Martinez, E. Mendioroz and M. Reinoso, *Int J Pharm*, 2007, **328**, 112–118.

6 C. Wang, I. Rosbottom, T. D. Turner, S. Laing, A. G. P. Maloney, A. Y. Sheikh, R. Docherty, Q. Yin and K. J. Roberts, *Pharm Res*, 2021, **38**, 971–990.

7 X. He, J. G. Stowell, K. R. Morris, R. R. Pfeiffer, H. Li, G. P. Stahly and S. R. Byrn, *Cryst Growth Des*, 2001, **1**, 305–312.

8 G. L. Perlovich, S. V. Blokhina, N. G. Manin, T. V. Volkova and V. V. Tkachev, *J Therm Anal Calorim*, 2013, **111**, 655–662.

9 J. C. Cole, O. Korb, P. McCabe, M. G. Read and R. Taylor, *J Chem Inf Model*, 2018, **58**, 615–629.

10 Y. Zhao and D. G. Truhlar, *Theor Chem Acc*, 2008, **120**, 215–241.

11 T. Schwabe and S. Grimme, *Physical Chemistry Chemical Physics*, 2007, **9**, 3397.

12 G. A. Petersson and M. A. Al-Laham, *J Chem Phys*, 1991, **94**, 6081–6090.

13 F. Weigend and R. Ahlrichs, *Physical Chemistry Chemical Physics*, 2005, **7**, 3297.

14 C. Greenwell and G. J. O. Beran, *Cryst Growth Des*, 2020, **20**, 4875–4881.
